# Molecular characteristics of odorant-binding protein 1 in *Anopheles maculipennis*

**DOI:** 10.1186/s12936-019-3058-6

**Published:** 2020-01-17

**Authors:** Mohammad Bagher Ghavami, Sakineh Khoeini, Navid Dinparast Djadid

**Affiliations:** 1 Department of Medical Entomology and Vector Control, School of Medicine, Zanjan University of Medical Sciences, Zanjan, Iran; 2 Malaria and Vector Research Group, Pasteur Institute of Iran, Tehran, Iran

**Keywords:** *Anopheles maculipennis*, Odorant-binding protein 1, *Amacobp1* gene

## Abstract

**Background:**

*Anopheles maculipennis* complex, the historic vector of malaria, causes serious medical problems worldwide and exhibits different behaviours. Studying the odorant-binding proteins (OBPs), which influence the chemosensory system and behavioural responses, is essential to understanding the population structure and developing effective control measures against this vector. The present study was designed to identify and analyse the *obp1* gene in *An. maculipennis.*

**Methods:**

Adults of *An. maculipennis* sensu stricto were collected in Zanjan Province, northwest of Iran, and gDNAs of female mosquitoes were extracted. Fragments of *An. maculipennis obp1* (*Amacobp1*) gene were amplified using degenerate and specific primers, and some of amplicons were selected for sequencing.

**Results:**

Analysis of amplified products identified that the sequence of *Amacobp1* gene was 1341 bp long. This gene contains three exons (5′, internal, and 3′of 160, 256, and 18 bp, respectively) and encodes 144 amino acids. The sizes of introns I and II in deduced gene are 268 and 358 nucleotides, respectively. The amino acid sequence in the C-terminal of AmacOBP1 is similar to that of major malaria vector *Anopheles* species. However, its N-terminal has a specific signal peptide with 19 amino acids. This peptide is conserved in different studied populations, and its sequence of amino acids shows the most variation among anopheline species.

**Conclusions:**

Degenerate primers in this study are suggested for studying *obp1* gene in *Anopheles* species. *Amacobp1* gene is proposed as a molecular marker for the detection of intraspecific ecotypes and diagnosis of different species within Maculipennis Group. Moreover, the N-terminal of *AmacOBP1* peptide is recommended as a molecular marker to identify the Amacobp1 expression patterns in different chemosensory organs for assessing the molecular mechanisms and developing novel behavioural disturbance agents to control *An. maculipennis*.

## Background

*Anopheles maculipennis* complex include the historic vectors of malaria in Holarctic regions and can cause serious public health problems worldwide [1–3]. In this complex, there are several sibling species, among which *An. maculipennis* sensu stricto (s.s.) is the dominant one in Europe and the Middle East regions [4–6]. These species occur partially sympatric and exhibit different behaviours in their host preference and selection of resting and breeding places [5–7]. Adults of these species are frequently found in human dwellings and animal shelters. They rest both indoor and outdoor shelters, and actively bite humans and animals [6–9]. Recently, various vector control methods have been developed against malaria vectors, such as *An. maculipennis*, and the most widely used method is insecticide application [10]. However, over the years, it has been reported that these species are resistant to some insecticides, such as DDT, organophosphate, carbamate, and pyrethroid [11, 12]. Accordingly, new mosquito-centered strategies for controlling and eliminating vector-borne diseases are urgently needed.

Repellents (impregnated bed nets and other formulations) seem to be applicable and efficient for the personal protection against biting insects and the diseases that they transmit to the inhabitants of endemic areas and travellers [10, 13]. These compounds interfere in the olfactory system of insects, thereby preventing the transmission and spread of vector-borne diseases [14, 15]. Knowledge about the molecular elements of the mosquito odorant detection system and their specific functions is crucial for developing novel and eco-friendly methods for mosquito control [16]. Although numerous studies have contributed to understanding structure of various genes and their application in differentiation of species in Maculipennis Group [5, 17–20], there is still lack of information on the olfactory system of these species. This information may provide new insight into understanding the mechanism governing the speciation within the aforesaid complex and separation of their ecotypes.

Odorant-binding proteins (OBPs) are the most important agents in the olfactory system. These proteins play key roles in odorant perception and transmission to receptor sites, signal transduction, and triggering a series of behavioural responses [14, 15]. The insect OBPs are soluble and globular peptides (15–40 kDa, ca 120–150 amino acids) with six highly cysteine-rich (Cys) residues, paired in three interlocked disulfide bridges [21, 22]. The affinities of these peptides for binding to ligand are pH-dependent [23]. The hydrogen bonds involving in the C-terminal loop break at low pH and open the loop, thus releasing further ligands [23–25]. These proteins are grouped into the following categories: classic, minus-C, plus-C, and atypical OBPs, based on the number of conserved Cys residues and their molecular structure [26]. Classic OBPs is the most important OBP in insects and contains six conserved cysteines having distinct disulfide connectives [26–28].

Among the classic group of the mentioned proteins, OBP1 is expressed exclusively in the head and olfactory organs of adult insects [29–31]. Meanwhile, the sexually-biased expression of this protein is exhibited in different species of insects [27, 29–34]. Moreover, this protein is expressed rhythmically, and its transcription level is reduced after blood feeding by less than twofold in mosquitoes and other blood-feeding insects [34, 35]. The three-dimensional structure of *Anopheles gambiae* OBP1 (AgamOBP1) has already been detected, and indole and DEET have been identified as its ligands [36–38]. The homolog to AgamOBP1 in *Anopheles stephensi* has also been found as AsteOBP1, and its expression has revealed that its transcription level is sevenfold higher in female’s than in male’s antenna [34]. The amino acid sequence of exon 1 in this gene has been suggested as a molecular marker for diagnosis of biological forms in *An. stephensi* [39]. Moreover, based on the nucleotide sequence variation of intron I *Asteobp1* gene in different populations, *An. stephensi* may prove to be a complex species [40].

Owing to the wide variation in the biological and ecological characteristics (host preference and vector competence.) within the members of Maculipennis Group, this study attempted to characterize, for the first time, the *An. maculipennis* OBP1 gene (*Amacobp1*), as a basic information for further studies to distinguish different taxa belonging to Maculipennis Group in the world. The present study also provides a new insight into the role of OBPs in the behaviour of different species and understanding speciation-related events within this complex.

## Methods

### Mosquito collection

Adults of *An. maculipennis* were collected by hand catch from Gheytoul (37°06′31.7″ N 47°47′28.9″ E), Ghara Bouta (37°03′31.3″ N 47°46′09.5″ E), Moshampa (36°56′33.8″ N 47°40′28.8″ E), Kheyrabad (36°55′25.4″ N 47°22′50.6″ E), and Sari Aghol (36°49′23.5″ N 47°37′46.8″ E) in Zanjan Province, the northwest of Iran, in summer 2016.

### Processing of mosquitoes

Samples of female mosquitoes were selected for molecular survey. Identification of *An. maculipennis* s.s. were carried out using morphological characters [3, 7] and PCR–RFLP assay, based on ITS2 ribosomal DNA [20]. To extract genomic DNA (gDNA), the samples were stored at − 70 °C for 1 h. Then the frozen samples were placed individually in 1.5-ml tubes and homogenized by an electrical homogenizer in a 400-µl TENS buffer (100 mM of Tris, pH 8.0, 10 mM of EDTA, 0.5 mM of NaCl, and 1% W/V SDS) containing a few glass beads. The gDNA of the samples was extracted as described by Ghavami et al. [20]. Briefly, 20 µg of proteinase K was added to each sample, mixed gently and incubated at 55 °C for 5 h. After incubation, 100 µl of 8 M potassium acetate was added and kept on ice bath for 10 min. The samples were then spun at 4000×g for 10 min, and the supernatants were transferred to new tubes. An equal volume of cold absolute ethanol was added to each tube, and the samples were spun at 8000×g for 10 min. Pellets of the samples were suspended in 0.5 ml of 70% cold ethanol and eventually spun at 8000×g for 10 min. The final pellet was dried at room temperature and re-suspended in 50 µl of TE buffer (10 mM Tris, pH 8.0, and 1 mM EDTA).

The PCR assays were conducted in two steps. In the first phase, the splicing region between the upstream and the downstream of deduced gene was amplified by degenerate primers. These primers were designed from the conserved regions of this gene in known anopheline species (Table 1) by means of BioEdit version 7 software [41]. These primers are not specific and could be used to generate the OBP1 gene in different anopheline species. The first stage of PCR was performed from mosquito gDNA in two separated ′a′ and ′b′ reactions. Each 50 µl of PCR reaction of this stage contained 4 µg of gDNA as the template, 1.5 mM of MgCl_2_, 0.2 mM of dNTPs (SinaClon, Iran), 10 pM of each primer (AnFa, AnRa and AnFb, AnRb; Table 2), and 1 U *Taq* of DNA polymerase (SinaClon, Iran). Thermal profile of PCR reaction was started with one cycle of initial denaturation at 95 °C for 3 min, followed by running through 30 cycles of 60 s at 94 °C, 60 s at 68 °C, and 90 s at 72 °C. The final extension was performed in one cycle at 72 °C for 5 min.

**Table 1:** Sequences of nucleotides in different genes of encoded odorant-binding protein 1 (OBP1) in *Anopheles* species used for designing the degenerate primers in this study

*Anopheles* species	Gene code^a^	Sequence location
*An. culicifacies*	ACUA014299	KI424042: 15893–17790
*An. dirus*	ADIR008409	KB672813: 2498551–2500404
*An. epiroticus*	AEPI007538	KB671606: 54036–56043
*An. farauti*	AFAF003898	KI915040: 6703195–6705104
*An. funestus*	AFUN008615	KB668715: 290624–291834
*An. gambiae*	AGAP029062	2R: 35643035–35644609
*An. melas*	AMEC013481	KI920369: 35115–36111
*An. merus*	AMEM011461	KI915244: 175565–177040
*An. quadriannulatus*	AQUA010363	KB665843: 2529565–2530556
*An. sinensis*	ASIS018762	KI916356: 352496–353910
*An. stephensi*	ASTE010148	KB664334: 168200–170200

^a^Gene numbers submitted to the VectorBase (https://www.vectorbase.org)

**Table 2:** Details of the primers used in this study

Primer name	Nucleotide sequence (5′ → 3′)
AnFa	TTGTCTCDTGATTGATTTGTCA
AnRa	CTTYTCGTCYTCGTGGATTCT
AnFb	AGATCCACGARGACGARAAG
AnRb	GAAAAATACGGTCTGATTATAG
AmaF	GTGTGGATAGTTCTTGGAACGG
AmaR	AAGAAATGCATCGCACACTCGC

R = A+G, Y = C+T, D = A+G

The second phase of PCR was performed by using the specific primers, AmaF and AmaR (Table 2). These primers, specific to *An. maculipennis* s.s., were designed based on the nucleotide sequence of the first PCR products. The forward primer (AmaF) was located around the translation start codon and the reverse primer (AmaR) at the translation stop of the *Amacobp1* gene. For each 50 µl of PCR reaction, 10 ng of gDNA was amplified with 10 pM of each primer using the following conditions: 95 °C for 5 min in one cycle, 93 °C for 30 s, 60 °C for 50 s, and 72 °C for 2 min. This cycle was repeated 35 times.

Each PCR run contained field samples and a negative control (molecular grade water instead of DNA template). Aliquots of the amplicons were analysed by electrophoresis on 1.5% agarose gel stained with safe stain (SinaClon, Iran) and visualized under a UV light. The amplified fragments were purified by PCR Clean-Up Kit (SinaClon, Iran) and sequenced bi-directionally by SeqLab GmbH (WWW.Seqlab.De) using the forward and reverse specific primers. The nucleotide sequences of the samples were aligned and edited by the aid of BioEdit and Sequin software (https://www.ncbi.nlm.nih.gov/Sequin). The *Amacobp1* nucleotide sequences in studied populations were submitted to the GenBank and are available with accession nos. MG209518-MG209522 and MN080505-MN080524.

The consensus of nucleotide and amino acid sequences were achieved by Basic Local Alignment Search Tool (BLAST) (https://blast.ncbi.nlm.nih.gov/Blast.cgi), BLASTn and P BLAST, which were searched for fragment identification and compared with other submitted relevant sequences in NCBI (https://www.ncbi.nlm.nih.gov), VectorBase (https://www.vectorbase.org) and FlyBase (http://www.flybase.org).

The mature peptide sequences identified in this study and in the previous studies were aligned using Clustal Omega (https://www.ebi.ac.uk/Tools/msa/clustalo/). The alignments were used to construct phylogenetic trees using the Molecular Evolutionary Genetics Analysis software version 6 (MEGA6) [42]. The final unrooted consensus trees were generated with 1000 bootstrap trials using the Neighbor-Joining, Maximum Likelihood, and Minimum-Evolution methods with a cut-off bootstrap value of 50 and using p-distance model.

## Results

**Fig. 1:**
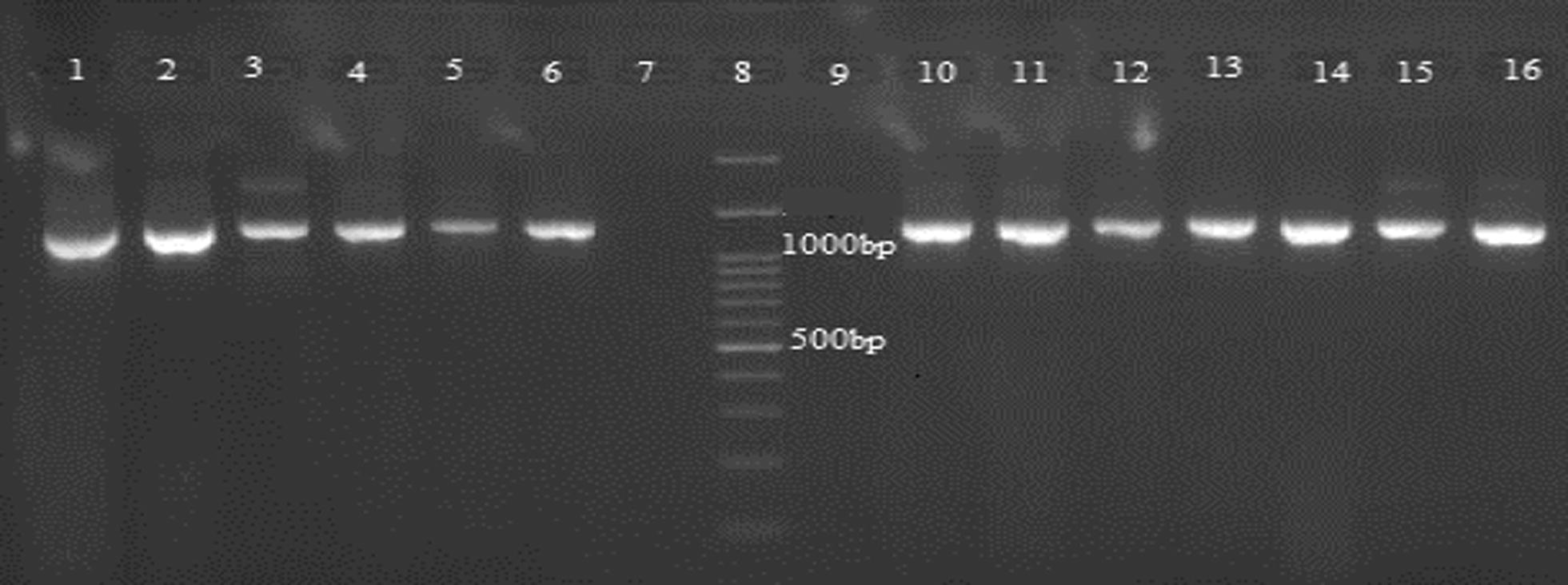
PCR amplification of *odorant*-*binding protein 1* fragment in *An. maculipennis* s. s. Lanes 1–3 and 4–6, the first and the second amplicons produced by degenerated primers, respectively; lanes 7 and 9, negative controls; lane 8, 100 bp DNA marker; lanes 10–16, products of specific primers

**Fig. 2:**
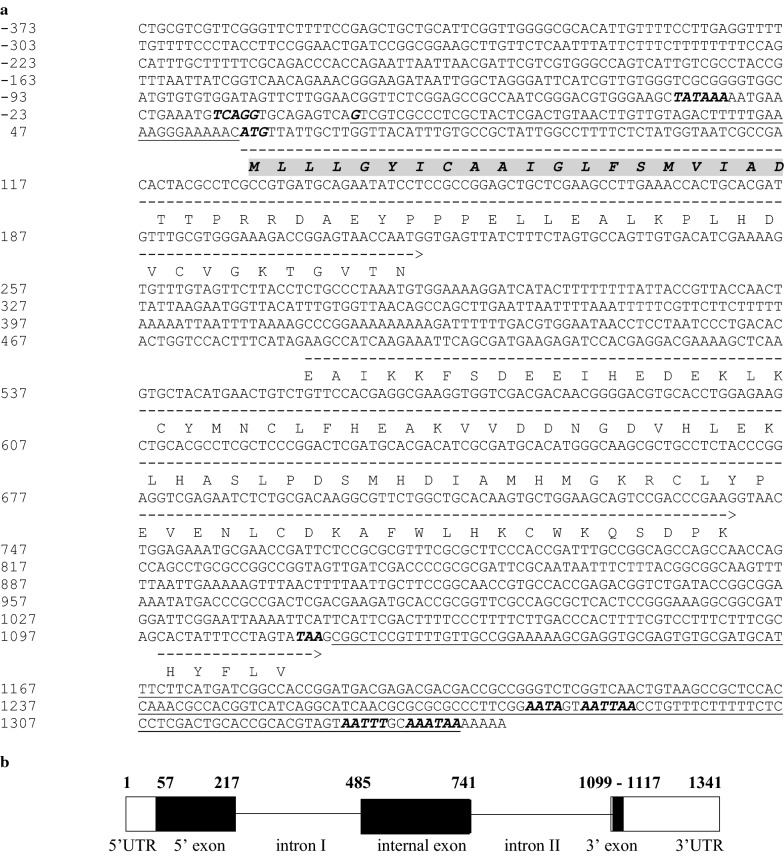
Sequence and schematic structure of *An. maculipennis* odorant-binding protein 1 (*Amacobp1*) gene. **a** Genomic sequence of *Amacobp1* gene containing two introns among three exons. The deduced amino acids sequences of *Amacobp1* gene is shown below the coding regions. Start (ATG) and stop (TAA) codons are italicized, in bold. A putative imitator sequence, TCAGG, similar to the arthropod imitator consensus, the consensus for TATA box/TATAAA, and the imperfect polyadenylation signals (AATA, AATTAA, AATTT, AAATAA) are shown in bold-italic text. Solid underlines indicate untranslated reading fragments (UTRs), and dashed underlines show the open reading fragments (ORFs) of exons. The signal peptide sequence is highlighted in gray and italicized. **b** Schematic structure of *Amacobp1* gene. 5′ exon, internal exon, and 3′ exon are indicated in boxes. Boxes with solid black show ORFs. They are connected with lines that indicate the introns. The 5′ and 3′UTRs are shown in hallow boxes. Numbers indicate the relative positions of UTRs, ORFs, and intron regions

The fragments of 1100 and a 1200 bp were amplified in the ‘a’ and ‘b’ section of the first PCR step, respectively, in 200 specimens (40 samples from each population) by degenerated primers. In the second step, a 1261-bp fragment was obtained in studied samples by specific primers (Fig. 1). A total of 20 PCR products of specific primers (4 samples from each population) and 5 PCR products of degenerated primers (one sample from each population) were selected randomly for sequencing. Analysing the sequences of degenerated primer products revealed that the sequence of *Amacobp1* gene has 1341 nucleotides. This analysis also exhibited the existence of two introns and three (5′, internal, and 3′) exons in deduced gene (Fig. 2). In amplified fragments, the transcription start site (GTCGTCG indicated as +1 in Fig. 2) occurred in 57 nucleotide upstream of the start codon (ATG). However, a TCAGG sequence, similar to the arthropod imitator, was observed in 15 nucleotide upstream of the transcription start site. The Goldberg-Hogness box, known as TATA box, is located in the 35-bp upstream of the predicted transcription start site. Imperfect polyadenylation signal sequences AATA, AATTAA, AATTT, and AAATAA were found in the 155, 161, 204, and 211 downstream of the poly (dA) tail, respectively. The position of transcription start site, the start and stop codons, and the open reading frame (ORF) was interrupted by two introns, and the putative polyadenylation signals are all shown in Fig. 2.

The processed transcript was 715 bp long with an approximately 57 bp 5′ UTR and 224 bp 3′ UTR. The coding region contained 434 bp long. In this region, three exons, including 5′, internal, and 3′ of 160, 256, and 18 bp, respectively, encoded 144 amino acids of OBP1. The sizes of intron I and intron II in the deduced gene were 268 and 358 nucleotides, respectively.

**Fig. 3:**
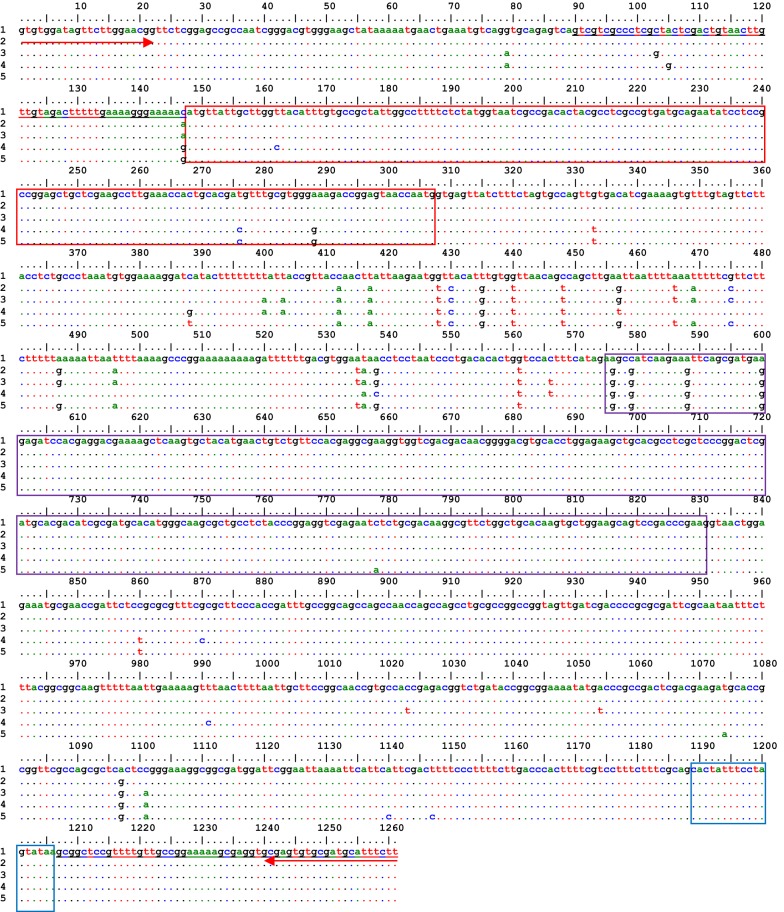
Nucleotide polymorphism and schematic structure of amplified fragment of *Amacobp1* gene in representative populations of *An. maculipennis* s.s. (based on 5 collected sites) with specific primers. Red arrows, indicate the position of specific primers. Horizontal numbers, indicate the study populations; 1: Kheyrabad (MG209521); 2: Ghara Bouta (MG209519); 3: Gheytoul (MG209520); 4: Sari Aghol (MG209518) and 5: Moshampa, (MG209522). UTRs are mentioned with underline letters, and 5′ exon, internal exon, and 3′ exon are shown in red, violet and blue boxes, respectively

Twenty fragments comprising 5′ UTR, ORF, and partial 3′ UTR of *Anmacobp1* gene were generated by specific primers. The alignment was 1261 bp in length (including primers 44 bp), and 41 sites (3%) were variable among the sequences. Each of 5′ and internal exons had three variable nucleotide sites, and the nucleotide polymorphism in introns I and II were in 12 and 10 sites, respectively. The percentages of nucleotide polymorphism in coding and noncoding regions of the deduced gene were 1.4% and 3.8%, respectively. All the single nucleotide polymorphisms in the coding region were silent. Multiple alignments of these sequences in representative populations are illustrated in Fig. 3.

**Fig. 4:**
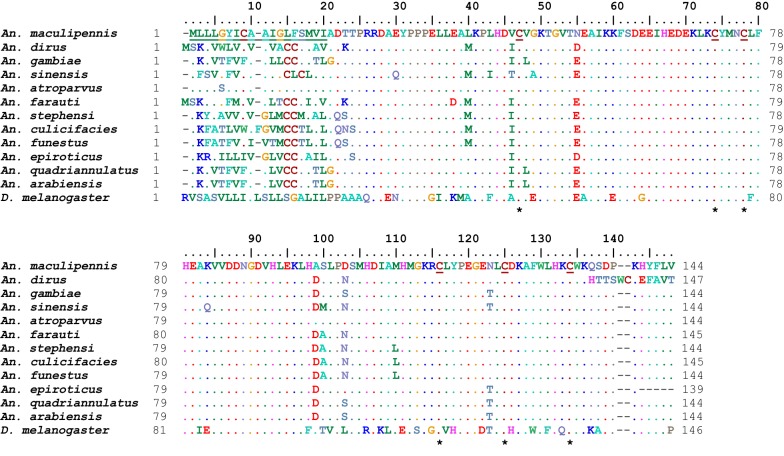
Alignment of *An. maculipennis* odorant-binding protein 1 (OBP1) amino acids with its homologue peptides in mosquitos and fruit flies. The six conserved cysteines are shown in underlined and in asterisks. Predicted signal peptide is underlined in the sequence. *An. dirus* (ADIR008409), *An. gambiae* (AGAP003309), *An. sinensis* (ASIS018762), *An. atroparvus* (AATE020811), *An. farauti* (AFAF003898), *An. stephensi* (ASTE010148), *An. culicifacies* (ACUA014299), *An. funestus* (AFUN008615), *An. epiroticus* (AEPI007538), *An. quadriannulatus* (AQUA010363), *An. arabiensis* (AARA001391) protein codes submitted to the VectorBase (http://www.vectorbase.org), and *D. melanogaster* (FBpp0078305) protein ID submitted to the FlyBase (http://www.flybase.org)

Standard protein BLAST analysis of *AmacOBP1* indicated that the overall structure of this peptide was highly similar to *Anopheles atroparvus* OBP1, which was annotated in VectorBase with AATE020811-RA code. The analysis also showed that *AmacOBP1* alignment shared 87% amino acid similarity with *Anopheles sinensis* (AIR09579), as well as 86% identity with *An. gambiae* (AAI84179), 83% with *An. stephensi* (ACS83757), and 83% with *Drosophila melanogaster* (FBpp0078305). Comparison of amino acid sequences of *AmacOBP1* with their homologous in *An. atroparvus* revealed that they had only one non-identical amino acid. In *An. maculipennis*, cysteine (C) had been replaced by glycine (G) (C → G) in locus 5 (Fig. 4).

Pairwise comparison of amino acids in OBP1 among different species of *Anopheles* showed the existence of six conserved C amino acids, five of which were in the first exon, and the remaining one in the second exon. This analysis also suggested that the two exons (II and III) were relatively conserved between anopheline species both in terms of their sequence and length. Comparing amino acid sequence of *AmacOBP1* with AgamOBP1 indicated that the C-terminal of *AmacOBP1* has a high similarity (95%, 3/60) to that of AgamOBP1.

The comparison between the N-terminal of *AmacOBP1* and its orthologous polypeptide in different species revealed that this region was less conserved, and 19 amino acids of this part were signal peptides and had specific sequencing. However, C-terminal of the *AmacOBP1* polypeptide had a high level of conservation between the species and exon III of deduced *obp1* gene in *An. maculipennis* encoding a short-chain amino acid peptide (6 amino acids), which its composition was similar to that of other anopheline species.

**Fig. 5:**
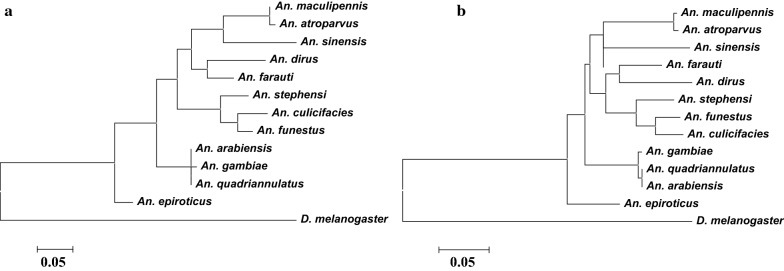
Phylogenetic tree relationship between *An. maculipennis* odorant-binding protein 1 (OBP1) with its homologues in *An. atroparvus* (AATE020811), *An. sinensis* (ASIS018762), *An. dirus* (ADIR008409), *An. farauti* (AFAF003898), *An. stephensi* (ASTE010148), *An. culicifacies* (ACUA014299), *An. funestus* (AFUN008615), *An. arabiensis* (AARA001391), *An. gambiae* (AGAP003309), *An. quadriannulatus* (AQUA010363), *An. epiroticus* (AEPI007538) protein codes submitted to the VectorBase (http://www.vectorbase.org), and *D. melanogaster* (FBpp0078305) protein ID submitted to the FlyBase (http://www.flybase.org). **a** Maximum likelihood; **b** neighbor-joining and minimum-evolution

The phylogenic relationship and topology based on the amino acid of polypeptide in deduced protein, along with sequences available in the VectorBase and FlyBase, is illustrated in Fig. 5. The Neighbour-Joining, Minimum Evolution, and Maximum likelihood analyses showed these OBPs cluster in three groups. *An. atroparus* and *An. sinensis* were the closest species to *An. maculipennis*. This clade, together with *Anopheles dirus* and *Anopheles farauti*, was deposited in one group. *Anopheles farauti*, *An. dirus*, *An. stephensi*, *Anopheles funestus*, and *Anopheles culicifacies* were sister groups with *An. maculipennis*. The remaining species, including *An. gambiae*, *Anopheles quadrimaculatus*, and *Anopheles arabiensis*, which were initially separated from anopheline species, were located in the third group (Fig. 5).

## Discussion

*Anopheles maculipennis* s.s. is the dominant anopheline species of the Maculipennis Group in Europe and the Middle East regions [4–9]. There has previously been no available information regarding *Amacobp* gene and sequence details of olfaction-associated genes in this species. Therefore, generating relevant genetic data was a research priority for investigations focusing on olfactory gene functions in different species of Maculipennis Group.

Using degenerate primers in this study, the fragment sequences of 1100 bp within gDNA, a region covering the *obp1* gene in *An. maculipennis*, were amplified by PCR, and, thus, these primers could be suggested for use in studying *obp1* gene in different *Anopheles* species. Applying degenerate primes to amplify the candidate *obp1* gene from gDNA is a convenient way to identify the molecular characters. Nonetheless, this method might miscalculate the data related to 5′ and 3′ UTRs of the gene. Hence, further studies are necessary to obtain the full-length cDNA clones from mRNA by RACE-PCR methods.

In the present study, analysis of amplified fragments, products with specific primers, showed that the ORF of *Amacobp1* gene has 1060 nucleotides, and this region encodes a 144-amino-acid polypeptide. Comparison of the amplified fragments in studied samples revealed that the *AmacOBP1* is conserved within the population level. To the best of our knowledge, this is the first report describing the *Amacobp1* gene. The generated information could be useful for the development of molecular techniques in studying *Amacobp1* expression patterns in different olfactory and gustatory organs.

Comparison of amino acid sequencing of *AmacOBP1* with its orthologous peptides demonstrated that this protein has 86% similarity with AgamOBP1. Previous studies have also indicated that AgamOBP1 displays high affinity with its ligands. The C-terminal loop of this peptide covers the binding cavity, and attractant and repellent have been identified as two of its ligands [36, 37]. The alignment of *AmacOBP1* with AgamOBP1 and other mosquito orthologous peptides showed a high level of conservation in the overall protein structure. The availability of the primary and the secondary structures of *AmacOBP1* would help prospective modelling studies that compare and contrast the two anopheline OBP1 proteins. Therefore, future studies are suggested to investigate the expression profile and ligand-binding feature in AmacOBP1. Moreover, identifying key amino acids in C-termini and detecting main hydrophobic, polar and nonpolar residues in signal peptides that involve in the ligand binding could be the other subjects for future investigations.

By comparing amino acid sequences in the C-terminal of *AmacOBP1* with its orthologous peptides a high identity (95%) was found between *AmacOBP1* and AgamOBP1. DEET and other repellents have been demonstrated to target OBP function by disrupting interaction with natural ligands to prevent connection with other compounds of the olfactory signaling pathway. Although effective, DEET has been recognized to be toxic, to have reduced efficacy with time after application, and to be ineffective against developed resistance species [43, 44]. Therefore, the need for a new, more efficient and less toxic repellent is highly felt. In silico ligand-binding studies in future may be valuable for screening mosquito repellents. In addition, X-ray crystallography and nuclear magnetic resonance (NMR) have successfully been applied to solve the structure of OBP from different insect species [24]. Comparative structure of *AmacOBP1* is certainly important in forthcoming research, particularly to address questions regarding molecular feature of the pH-dependent conformation changes (Additional file 1).

The phylogeny analysis in the present study showed a great variety in the amino acid alignment of OBP1 peptides in different *Anopheles* species, and *AmacOBP1* exhibited the highest amino acid variation among these peptides. Considering these results, it is suggested that the OBP1-encoding gene in the anopheline is derived from the same ancestor and diverged after speciation. On the other hand, the *AmacOBP1*, which has the most variation, may have a long history or high rate of evolution in adaptation to ecological conditions. The high rate of variation within *AmacOBP1* may suggest the ongoing evolutionary mechanisms, including genetic drift and gene flow that are still faster in the speciation within this complex. This observation may be explained in further studies by the wide variation and distribution patterns of different members in the Maculipennis Group.

The present study showed that the N-terminal of *AmacOBP1* assets signal peptides. Despite the consistency of amino acid sequences in C-terminal, this part has specific sequence and little sequence conservation. In the context of evolution, the pressures that shaped the properties of the mature OBP proteins are fundamentally different from those that shaped their signal peptides. The mature proteins evolve to interact with external chemical signals and to function in a behavioural context, while the signal peptides develop to interact with intracellular signals involved in trafficking. All of these OBPs are postulated to be expressed in and secreted by glia-like support cells that ensheathe the sensory neuron somata and have highly folded apical membranes facing the sensillum lumen. The OBPs are presumably secreted across these apical membranes. The OBP signal peptides contain the hydrophobic cores of 10–12 amino acids flanked by more hydrophilic regions. These characteristics of signal peptides are involved in protein translocation into endoplasmic reticulum for the purpose of secretion [45, 46]. In the same general cell type, these intracellular signals are expected to be conserved, especially within a given mosquito species, and are poorly conserved between species. This class of conservation suggests that OBP1 signal peptides might be involved in class-specific processing for expressed homologous proteins. Therefore, they could be used as a molecular marker for studying the population structure of species in Maculipennis Group. Moreover, signal peptides are appropriate sequences to design specific primers and probes for studying the gene expression pattern in olfactory and gustatory organs of *An. maculipennis* by qPCR assay in different populations.

## Conclusion

Degenerate primers in this research are suggested for study on *obp1* gene in different *Anopheles* species. Prospective studies are recommended to investigate the gene expression profile and ligand-binding profiles in *AmacOBP1*. Further investigations are also suggested to identify the key amino acids, main hydrophobic and polar/nonpolar residues of *AmacOBP1* C-terminal in ligand-binding. The signal peptide in N-terminal of *AmacOBP1* is advised for designing specific primers and probes, to detect the expression pattern of this gene in the main olfactory and gustatory organs for assessing the molecular mechanisms and developing novel repellents against this vector in future surveys. In addition, this peptide is proposed as a molecular marker for detection of *An. maculipennis* intraspecific ecotypes and diagnosis of different species within Maculipennis Group.

## Supplementary information

**Additional file 1: Appendix 1.** Nucleotide polymorphism and schematic structure of amplified fragment of *Amacobp1* gene in 25 samples of studied populations of *An. maculipennis* s. s.

## Data Availability

Data supporting of this article is included within the article and additional file.

## Abbreviations

AmacOBP1: *Anopheles maculipennis* odorant-binding protein 1
BLAST: basic local alignment search tool
BLASTn: basic local alignment search tool for nucleotides
bp: base pair
DDT: dichlorodiphenyltrichloroethane
DEET: N, N-diethyl-m-toluamide
gDNA: genomic DNA
ITS2: internal transcript spacer 2
kD: Kilo Dalton
MEGA6: molecular evolutionary genetics analysis version 6
mRNA: messenger ribonucleic acid
NCBI: The National Center for Biotechnology Information
OBPs: odorant-binding proteins
ORF: open reading frame
P BLAST: basic local alignment search tool for peptides
PCR: polymerase chain reaction
RACE: rapid amplification of cDNA ends
RFLP: restriction fragment length polymorphism
s.s.: sensu stricto
UTR: untranslated region
