# Supplementary material for: Molecular characteristics of odorant-binding protein 1 in Anopheles maculipennis
Source: Malar J. 2020 Jan 17;19:29. doi: 10.1186/s12936-019-3058-6 (PMC6969430; doi:10.1186/s12936-019-3058-6)
Supplement: Supplementary file 1 — Additional file 1: Appendix 1. Nucleotide polymorphism and schematic structure of amplified fragment of Amacobp1 gene in 25 samples of studied populations of An. maculipennis s. s. [file 12936_2019_3058_MOESM1_ESM.rtf]

                   10        20        30        40        50        60        70        80        90 
          ....|....|....|....|....|....|....|....|....|....|....|....|....|....|....|....|....|....|
IRZN-KH2  gtgtggatagttcttggaacggttctcggagccgccaatcgggacgtgggaagctataaaaatgaactgaaatgtcaggtgcagagtcag 
IRZN-KH3  .......................................................................................... 
IRZN-KH4  ..............................................................................a........... 
IRZN-KH5  .......................................................................................... 
IRZN-GB2  ..............................................................................a........... 
IRZN-GB3  .......................................................................................... 
IRZN-GB4  .......................................................................................... 
IRZN-GB5  .......................................................................................... 
IRZN-GT2  ..............................................................................a........... 
IRZN-GT3  ..............................................................................a........... 
IRZN-GT4  .......................................................................................... 
IRZN-GT5  ..............................................................................a........... 
IRZN-SA2  ..............................................................................a........... 
IRZN-SA3  ..............................................................................a........... 
IRZN-SA4  .......................................................................................... 
IRZN-SA5  ..............................................................................a........... 
IRZN-MS2  .......................................................................................... 
IRZN-MS3  .......................................................................................... 
IRZN-MS4  ..............................................................................a........... 
IRZN-MS5  .......................................................................................... 
IRZN-GB1  .......................................................................................... 
IRZN-GT1  ..............................................................................a........... 
IRZN-SA1  ..............................................................................a........... 
IRZN-MS1  .......................................................................................... 
IRZN-KH1  .......................................................................................... 

                  100       110       120       130       140       150       160       170       180 
          ....|....|....|....|....|....|....|....|....|....|....|....|....|....|....|....|....|....|
IRZN-KH2  tcgtcgccctcgctactcgactgtaacttgttgtagactttttgaaaagggaaaaacatgttattgcttggttacatttgtgccgctatt 
IRZN-KH3  .......................................................................................... 
IRZN-KH4  .......................................................................................... 
IRZN-KH5  .......................................................................................... 
IRZN-GB2  ............g...........................................a................................. 
IRZN-GB3  ........................................................a................................. 
IRZN-GB4  ..............g.........................................a................................. 
IRZN-GB5  ........................................................a................................. 
IRZN-GT2  ............g...........................................a................................. 
IRZN-GT3  ........................................................a................................. 
IRZN-GT4  ............g...........................................a................................. 
IRZN-GT5  ............g...........................................a................................. 
IRZN-SA2  ..............g.........................................g..............c.................. 
IRZN-SA3  ..............c.........................................g..............c.................. 
IRZN-SA4  ............g.g.........................................g..............c.................. 
IRZN-SA5  ..............g.........................................g..............c.................. 
IRZN-MS2  ........................................................g................................. 
IRZN-MS3  ........................................................g................................. 
IRZN-MS4  ..............g.........................................g................................. 
IRZN-MS5  ........................................................g................................. 
IRZN-GB1  ........................................................a................................. 
IRZN-GT1  ............g...........................................a................................. 
IRZN-SA1  ..............g.........................................g..............c.................. 
IRZN-MS1  ........................................................g................................. 
IRZN-KH1  .......................................................................................... 

                  190       200       210       220       230       240       250       260       270        
          ....|....|....|....|....|....|....|....|....|....|....|....|....|....|....|....|....|....|
IRZN-KH2  ggccttttctctatggtaatcgccgacactacgcctcgccgtgatgcagaatatcctccgccggagctgctcgaagccttgaaaccactg 
IRZN-KH3  .......................................................................................... 
IRZN-KH4  .......................................................................................... 
IRZN-KH5  .......................................................................................... 
IRZN-GB2  .......................................................................................... 
IRZN-GB3  .......................................................................................... 
IRZN-GB4  .......................................................................................... 
IRZN-GB5  .......................................................................................... 
IRZN-GT2  .......................................................................................... 
IRZN-GT3  .......................................................................................... 
IRZN-GT4  .......................................................................................... 
IRZN-GT5  .......................................................................................... 
IRZN-SA2  .......................................................................................... 
IRZN-SA3  .......................................................................................... 
IRZN-SA4  .......................................................................................... 
IRZN-SA5  .......................................................................................... 
IRZN-MS2  .....................a.................................................................... 
IRZN-MS3  .....................a.................................................................... 
IRZN-MS4  .....................a.................................................................... 
IRZN-MS5  .....................a.................................................................... 
IRZN-GB1  .......................................................................................... 
IRZN-GT1  .......................................................................................... 
IRZN-SA1  .......................................................................................... 
IRZN-MS1  .....................a.................................................................... 
IRZN-KH1  .......................................................................................... 

                  280       290       300       310       320       330       340       350       360        
          ....|....|....|....|....|....|....|....|....|....|....|....|....|....|....|....|....|....|
IRZN-KH2  cacgatgtttgcgtgggaaagaccggagtaaccaatggtgagttatctttctagtgccagttgtgacatcgaaaagtgtttgtagttctt 
IRZN-KH3  .......................................................................................... 
IRZN-KH4  .......................................................................................... 
IRZN-KH5  .......................................................................................... 
IRZN-GB2  .......................................................................................... 
IRZN-GB3  .......................................................................................... 
IRZN-GB4  .......................................................................................... 
IRZN-GB5  .......................................................................................... 
IRZN-GT2  .......................................................................................... 
IRZN-GT3  .......................................................................................... 
IRZN-GT4  .......................................................................................... 
IRZN-GT5  .......................................................................................... 
IRZN-SA2  .....c...........g............................................t........................... 
IRZN-SA3  .....c...........g............................................t........................... 
IRZN-SA4  .....c...........g............................................t........................... 
IRZN-SA5  .....c...........g............................................t........................... 
IRZN-MS2  .....c...........g............................................t........................... 
IRZN-MS3  .....c...........g............................................t........................... 
IRZN-MS4  .....c...........g............................................t........................... 
IRZN-MS5  .....c...........g............................................t........................... 
IRZN-GB1  .......................................................................................... 
IRZN-GT1  .......................................................................................... 
IRZN-SA1  .....c...........g............................................t........................... 
IRZN-MS1  .....c...........g............................................t........................... 
IRZN-KH1  .......................................................................................... 

                  370       380       390       400       410       420       430       440       450        
          ....|....|....|....|....|....|....|....|....|....|....|....|....|....|....|....|....|....|
IRZN-KH2  acctctgccctaaatgtggaaaaggatcatacttttttttattaccgttaccaacttattaagaatggttacatttgtggttaacagcca 
IRZN-KH3  .....................................................................c....g............... 
IRZN-KH4  ...................................................a...............t...................t.. 
IRZN-KH5  .......................................................................................... 
IRZN-GB2  ...................................................a....a..........t.c....g....t.......t.. 
IRZN-GB3  ...................................................a....a..........t......g....t.......t.. 
IRZN-GB4  ...................................................a.................c....g....t.......t.. 
IRZN-GB5  ........................................................a..........t.c.................t.. 
IRZN-GT2  .......................................a..a........a....a..........t.c....g....t.......... 
IRZN-GT3  .......................................a..a........a....a..........t......g....t.......t.. 
IRZN-GT4  .......................................a..a........a.................c....g....t.......t.. 
IRZN-GT5  .......................................a..a.............a..........t.c.........t.......t.. 
IRZN-SA2  ...........................g...........a..a........a....a..........t.c....g....t.......... 
IRZN-SA3  ...........................g...........a..a........a....a.................g............t.. 
IRZN-SA4  ...........................g...........a..a........a...............t.c....g....t.......t.. 
IRZN-SA5  ...........................g...........a..a.............a..........t.c.........t.......t.. 
IRZN-MS2  ...........................t.......................a....a..........t......g....t.......... 
IRZN-MS3  ...........................t.......................a....a............c....g....t.......t.. 
IRZN-MS4  ...........................t.......................a...............t.c....g............t.. 
IRZN-MS5  ...........................t............................a..........t...........t.......... 
IRZN-GB1  ...................................................a....a..........t.c....g....t.......t.. 
IRZN-GT1  .......................................a..a........a....a..........t.c....g....t.......t.. 
IRZN-SA1  ...........................g...........a..a........a....a..........t.c....g....t.......t.. 
IRZN-MS1  ...........................t.......................a....a..........t.c....g....t.......t.. 
IRZN-KH1  .......................................................................................... 

                  460       470       480       490       500       510       520       530       540        
          ....|....|....|....|....|....|....|....|....|....|....|....|....|....|....|....|....|....|
IRZN-KH2  gcttgaattaattttaaatttttcgttcttctttttaaaaattaattttaaaagcccggaaaaaaaaagattttttgacgtggaataacc 
IRZN-KH3  .......................................................................................... 
IRZN-KH4  .......................................................................................... 
IRZN-KH5  .......................................................................................... 
IRZN-GB2  ......g........t..a.....c...........g........a......................................ta.g.. 
IRZN-GB3  ...............t..a.....c............................................................a.g.. 
IRZN-GB4  ......g.............................g........a......................................ta.... 
IRZN-GB5  ......g........t..a.....c...........g........a......................................ta.g.. 
IRZN-GT2  ......g........t..a.....c...........g........a......................................ta.g.. 
IRZN-GT3  ...............t..a..........................a.......................................a.g.. 
IRZN-GT4  ......g........t..a.....c...........g...............................................ta.... 
IRZN-GT5  ......g........t........c...........g........a......................................ta.g.. 
IRZN-SA2  ......t..............................................................................a.c.. 
IRZN-SA3  ......t..............................................................................a.... 
IRZN-SA4  ......t..............................................................................a.c.. 
IRZN-SA5  ......t..............................................................................a.c.. 
IRZN-MS2  ......g........t..a.....c...........g........a......................................ta.g.. 
IRZN-MS3  ......g........t..a.................................................................ta.... 
IRZN-MS4  ........................c...........g........a.......................................a.g.. 
IRZN-MS5  ......g........t..a.....c...........g........a......................................ta.g.. 
IRZN-GB1  ......g........t..a.....c...........g........a......................................ta.g.. 
IRZN-GT1  ......g........t..a.....c...........g........a......................................ta.g.. 
IRZN-SA1  ......t..............................................................................a.c.. 
IRZN-MS1  ......g........t..a.....c...........g........a......................................ta.g.. 
IRZN-KH1  .......................................................................................... 

                  550       560       570       580       590       600       610       620       630        
          ....|....|....|....|....|....|....|....|....|....|....|....|....|....|....|....|....|....|
IRZN-KH2  tcctaatccctgacacactggtccactttcatagaagccatcaagaaattcagcgatgaagagatccacgaggacgaaaagctcaagtgc 
IRZN-KH3  ...................................g...................................................... 
IRZN-KH4  ....................t.................g........g...........g.............................. 
IRZN-KH5  .......................................................................................... 
IRZN-GB2  ...................................g..g........g...........g.............................. 
IRZN-GB3  ....................t..............g...........g...........g.............................. 
IRZN-GB4  ....................t.................g........g.......................................... 
IRZN-GB5  ....................t..............g..g........g...........g.............................. 
IRZN-GT2  ....................t....t.........g..g........g...........g.............................. 
IRZN-GT3  ....................t....t............g....................g.............................. 
IRZN-GT4  ...................................g...........g...........g.............................. 
IRZN-GT5  ....................t....t.........g..g........g.......................................... 
IRZN-SA2  ....................t....t.........g...........g...........g.............................. 
IRZN-SA3  ....................t..............g..g........g...........g.............................. 
IRZN-SA4  .........................t............g....................g.............................. 
IRZN-SA5  ....................t....t.........g..g........g.......................................... 
IRZN-MS2  ....................t..............g..g........g...........g.............................. 
IRZN-MS3  ....................t..............g...........g...........g.............................. 
IRZN-MS4  ...................................g..g....................g.............................. 
IRZN-MS5  ....................t.................g........g.......................................... 
IRZN-GB1  ....................t..............g..g........g...........g.............................. 
IRZN-GT1  ....................t....t.........g..g........g...........g.............................. 
IRZN-SA1  ....................t....t.........g..g........g...........g.............................. 
IRZN-MS1  ....................t..............g..g........g...........g.............................. 
IRZN-KH1  .......................................................................................... 

                  640       650       660       670       680       690       700       710       720        
          ....|....|....|....|....|....|....|....|....|....|....|....|....|....|....|....|....|....|
IRZN-KH2  tacatgaactgtctgttccacgaggcgaaggtggtcgacgacaacggggacgtgcacctggagaagctgcacgcctcgctcccggactcg 
IRZN-KH3  .......................................................................................... 
IRZN-KH4  .......................................................................................... 
IRZN-KH5  .......................................................................................... 
IRZN-GB2  .......................................................................................... 
IRZN-GB3  .......................................................................................... 
IRZN-GB4  .......................................................................................... 
IRZN-GB5  .......................................................................................... 
IRZN-GT2  .......................................................................................... 
IRZN-GT3  .......................................................................................... 
IRZN-GT4  .......................................................................................... 
IRZN-GT5  .......................................................................................... 
IRZN-SA2  .......................................................................................... 
IRZN-SA3  .......................................................................................... 
IRZN-SA4  .......................................................................................... 
IRZN-SA5  .......................................................................................... 
IRZN-MS2  .......................................................................................... 
IRZN-MS3  .......................................................................................... 
IRZN-MS4  .......................................................................................... 
IRZN-MS5  .......................................................................................... 
IRZN-GB1  .......................................................................................... 
IRZN-GT1  .......................................................................................... 
IRZN-SA1  .......................................................................................... 
IRZN-MS1  .......................................................................................... 
IRZN-KH1  .......................................................................................... 

                  730       740       750       760       770       780       790       800       810        
          ....|....|....|....|....|....|....|....|....|....|....|....|....|....|....|....|....|....|
IRZN-KH2  atgcacgacatcgcgatgcacatgggcaagcgctgcctctacccggaggtcgagaatctctgcgacaaggcgttctggctgcacaagtgc 
IRZN-KH3  .......................................................................................... 
IRZN-KH4  .......................................................................................... 
IRZN-KH5  .......................................................................................... 
IRZN-GB2  .......................................................................................... 
IRZN-GB3  .......................................................................................... 
IRZN-GB4  .......................................................................................... 
IRZN-GB5  .......................................................................................... 
IRZN-GT2  .......................................................................................... 
IRZN-GT3  .......................................................................................... 
IRZN-GT4  .......................................................................................... 
IRZN-GT5  .......................................................................................... 
IRZN-SA2  .......................................................................................... 
IRZN-SA3  .......................................................................................... 
IRZN-SA4  .......................................................................................... 
IRZN-SA5  .......................................................................................... 
IRZN-MS2  .........................................................a................................ 
IRZN-MS3  .........................................................a................................ 
IRZN-MS4  .........................................................a................................ 
IRZN-MS5  .........................................................a................................ 
IRZN-GB1  .......................................................................................... 
IRZN-GT1  .......................................................................................... 
IRZN-SA1  .......................................................................................... 
IRZN-MS1  .........................................................a................................ 
IRZN-KH1  .......................................................................................... 

                  820       830       840       850       860       870       880       890       900        
          ....|....|....|....|....|....|....|....|....|....|....|....|....|....|....|....|....|....|
IRZN-KH2  tggaagcagtccgacccgaaggtaactggagaaatgcgaaccgattctccgcgcgtttcgcgcttcccaccgatttgccggcagccagcc 
IRZN-KH3  .......................................................................................... 
IRZN-KH4  .......................................................................................... 
IRZN-KH5  .......................................................................................... 
IRZN-GB2  .......................................................................................... 
IRZN-GB3  .......................................................................................... 
IRZN-GB4  .......................................................................................... 
IRZN-GB5  .......................................................................................... 
IRZN-GT2  .......................................................................................... 
IRZN-GT3  .......................................................................................... 
IRZN-GT4  .......................................................................................... 
IRZN-GT5  .......................................................................................... 
IRZN-SA2  .................................................t.........c.............................. 
IRZN-SA3  .................................................t.........c.............................. 
IRZN-SA4  .................................................t.........c.............................. 
IRZN-SA5  .................................................t.........c.............................. 
IRZN-MS2  .................................................t........................................ 
IRZN-MS3  .................................................t........................................ 
IRZN-MS4  .................................................t........................................ 
IRZN-MS5  .................................................t........................................ 
IRZN-GB1  .......................................................................................... 
IRZN-GT1  .......................................................................................... 
IRZN-SA1  .................................................t.........c.............................. 
IRZN-MS1  .................................................t........................................ 
IRZN-KH1  .......................................................................................... 

                  910       920       930       940       950       960       970       980       990        
          ....|....|....|....|....|....|....|....|....|....|....|....|....|....|....|....|....|....|
IRZN-KH2  aaccagccagcctgcgccggccggtagttgatcgaccccgcgcgattcgcaataatttctttacggcggcaagtttttaattgaaaaagt 
IRZN-KH3  .......................................................................................... 
IRZN-KH4  .......................................................................................... 
IRZN-KH5  .......................................................................................... 
IRZN-GB2  .......................................................................................... 
IRZN-GB3  .......................................................................................... 
IRZN-GB4  .......................................................................................... 
IRZN-GB5  .......................................................................................... 
IRZN-GT2  .......................................................................................... 
IRZN-GT3  .......................................................................................... 
IRZN-GT4  .......................................................................................... 
IRZN-GT5  .......................................................................................... 
IRZN-SA2  .......................................................................................... 
IRZN-SA3  .......................................................................................... 
IRZN-SA4  .......................................................................................... 
IRZN-SA5  .......................................................................................... 
IRZN-MS2  .......................................................................................... 
IRZN-MS3  .......................................................................................... 
IRZN-MS4  .......................................................................................... 
IRZN-MS5  .......................................................................................... 
IRZN-GB1  .......................................................................................... 
IRZN-GT1  .......................................................................................... 
IRZN-SA1  .......................................................................................... 
IRZN-MS1  .......................................................................................... 
IRZN-KH1  .......................................................................................... 

                  1000      1010      1020      1030      1040      1050      1060      1070      1080       
          ....|....|....|....|....|....|....|....|....|....|....|....|....|....|....|....|....|....|
IRZN-KH2  ttaacttttaattgcttccggcaaccgtgccaccgagacggtctgataccggcggaaaatatgacccgccgactcgacgaagatgcaccg 
IRZN-KH3  .......................................................................................... 
IRZN-KH4  .......................................................................................... 
IRZN-KH5  .......................................................................................... 
IRZN-GB2  .......................................................................................... 
IRZN-GB3  .......................................................................................... 
IRZN-GB4  .......................................................................................... 
IRZN-GB5  .......................................................................................... 
IRZN-GT2  ................................t..............................t.......................... 
IRZN-GT3  ................................t..............................t.......................... 
IRZN-GT4  ................................t..............................t.......................... 
IRZN-GT5  ................................t..............................t.......................... 
IRZN-SA2  c......................................................................................... 
IRZN-SA3  c......................................................................................... 
IRZN-SA4  c......................................................................................... 
IRZN-SA5  c......................................................................................... 
IRZN-MS2  ...................................................................................a...... 
IRZN-MS3  ...................................................................................a...... 
IRZN-MS4  ...................................................................................a...... 
IRZN-MS5  ...................................................................................a...... 
IRZN-GB1  .......................................................................................... 
IRZN-GT1  ................................t..............................t.......................... 
IRZN-SA1  c......................................................................................... 
IRZN-MS1  ...................................................................................a...... 
IRZN-KH1  .......................................................................................... 

                  1090      1100      1110      1120      1130      1140      1150      1160      1170       
          ....|....|....|....|....|....|....|....|....|....|....|....|....|....|....|....|....|....|
IRZN-KH2  cggttcgccagcgctcactccgggaaaggcggcgatggattcggaattaaaattcattcattcgacttttcccttttcttgacccacttt 
IRZN-KH3  ................g......................................................................... 
IRZN-KH4  .......................................................................................... 
IRZN-KH5  .......................................................................................... 
IRZN-GB2  ................g...a..................................................................... 
IRZN-GB3  ................g......................................................................... 
IRZN-GB4  ................g......................................................................... 
IRZN-GB5  .......................................................................................... 
IRZN-GT2  ................g...a..................................................................... 
IRZN-GT3  ................g...a..................................................................... 
IRZN-GT4  ................g...a..................................................................... 
IRZN-GT5  ....................a..................................................................... 
IRZN-SA2  ................g......................................................................... 
IRZN-SA3  ................g...a..................................................................... 
IRZN-SA4  ................g...a..................................................................... 
IRZN-SA5  ....................a..................................................................... 
IRZN-MS2  ................g..........................................c......c....................... 
IRZN-MS3  ................g...a......................................c.............................. 
IRZN-MS4  ....................a......................................c......c....................... 
IRZN-MS5  ................g.................................................c....................... 
IRZN-GB1  ................g......................................................................... 
IRZN-GT1  ................g...a..................................................................... 
IRZN-SA1  ................g...a..................................................................... 
IRZN-MS1  ................g...a......................................c......c....................... 
IRZN-KH1  .......................................................................................... 

                  1180      1190      1200      1210      1220      1230      1240      1250      1260       
          ....|....|....|....|....|....|....|....|....|....|....|....|....|....|....|....|....|....|
IRZN-KH2  tcgtcctttctttcgcagcactatttcctagtataagcggctccgttttgttgccggaaaaagcgaggtgcgagtgtgcgatgcatttct 
IRZN-KH3  .......................................................................................... 
IRZN-KH4  .......................................................................................... 
IRZN-KH5  .......................................................................................... 
IRZN-GB2  .......................................................................................... 
IRZN-GB3  .......................................................................................... 
IRZN-GB4  .......................................................................................... 
IRZN-GB5  .......................................................................................... 
IRZN-GT2  .......................................................................................... 
IRZN-GT3  .......................................................................................... 
IRZN-GT4  .......................................................................................... 
IRZN-GT5  .......................................................................................... 
IRZN-SA2  .......................................................................................... 
IRZN-SA3  .......................................................................................... 
IRZN-SA4  .......................................................................................... 
IRZN-SA5  .......................................................................................... 
IRZN-MS2  .......................................................................................... 
IRZN-MS3  .......................................................................................... 
IRZN-MS4  .......................................................................................... 
IRZN-MS5  .......................................................................................... 
IRZN-GB1  .......................................................................................... 
IRZN-GT1  .......................................................................................... 
IRZN-SA1  .......................................................................................... 
IRZN-MS1  .......................................................................................... 
IRZN-KH1  .......................................................................................... 

          
          .
IRZN-KH2  t 
IRZN-KH3  . 
IRZN-KH4  . 
IRZN-KH5  . 
IRZN-GB2  . 
IRZN-GB3  . 
IRZN-GB4  . 
IRZN-GB5  . 
IRZN-GT2  . 
IRZN-GT3  . 
IRZN-GT4  . 
IRZN-GT5  . 
IRZN-SA2  . 
IRZN-SA3  . 
IRZN-SA4  . 
IRZN-SA5  . 
IRZN-MS2  . 
IRZN-MS3  . 
IRZN-MS4  . 
IRZN-MS5  . 
IRZN-GB1  . 
IRZN-GT1  . 
IRZN-SA1  . 
IRZN-MS1  . 
IRZN-KH1  . 


Fig A Nucleotide polymorphism and schematic structure of amplified fragment of Amacobp1 gene in 25 samples of studied populations of An. maculipennis s s.
